# Safety of resident training in the microsurgical resection of intracranial tumors: Data from a prospective registry of complications and outcome

**DOI:** 10.1038/s41598-018-37533-3

**Published:** 2019-01-30

**Authors:** Flavio Vasella, Julia Velz, Marian C. Neidert, Stephanie Henzi, Johannes Sarnthein, Niklaus Krayenbühl, Oliver Bozinov, Luca Regli, Martin N. Stienen

**Affiliations:** 1 University Hospital Zurich, Department of Neurosurgery, Zurich, Switzerland; 2 Clinical Neuroscience Center, University of Zurich, Zurich, Switzerland

## Abstract

The aim of the present study was to assess the safety of microsurgical resection of intracranial tumors performed by supervised neurosurgical residents. We analyzed prospectively collected data from our institutional patient registry and dichotomized between procedures performed by supervised neurosurgery residents (defined as teaching procedures) or board-certified faculty neurosurgeons (defined as non-teaching procedures). The primary endpoint was morbidity at discharge, defined as a postoperative decrease of ≥10 points on the Karnofsky Performance Scale (KPS). Secondary endpoints included 3-month (M3) morbidity, mortality, the in-hospital complication rate, and complication type and severity. Of 1,446 consecutive procedures, 221 (15.3%) were teaching procedures. Patients in the teaching group were as likely as patients in the non-teaching group to experience discharge morbidity in both uni- (OR 0.85, 95%CI 0.60–1.22, p = 0.391) and multivariate analysis (adjusted OR 1.08, 95%CI 0.74–1.58, p = 0.680). The results were consistent at time of the M3 follow-up and in subgroup analyses. In-hospital mortality was equally low (0.24 vs. 0%, p = 0.461) and the likelihood (p = 0.499), type (p = 0.581) and severity of complications (p = 0.373) were similar. These results suggest that microsurgical resection of carefully selected intracranial tumors can be performed safely by supervised neurosurgical residents without increasing the risk of morbidity, mortality or perioperative complications. Appropriate allocation of operations according to case complexity and the resident’s experience level, however, appears essential.

## Introduction

Training residents in surgical specialties while maintaining the best quality of patient care can be challenging. It is obvious that structured residency programs in surgical specialties should ideally provide residents with ample opportunities to perform operations in order to prepare them for board certification and the responsibilities of an attending physician. However, the question often arises, whether this necessary training process comes at the cost of inferior patient outcomes or an increased perioperative complication rate.

In recent years, a body of accumulating pro- and retrospective data indicates that supervised surgical training of residents is safe for some basic spinal and cranial procedures in terms of complication rates and outcomes^1–8^. While the previous articles imply that the lower experience level did not adversely affect patients undergoing relatively simple neurosurgical procedures such as burr hole trepanation^7^ or ventriculo-peritoneal shunt implantation^8^, this must not necessarily be true for technically more challenging procedures. In order to perform maximum safe microsurgical resection of intracranial tumors, for example, the trainee must have obtained considerable amounts of knowledge and practical skills. These include in-depth comprehension of functional anatomy of the brain, surgical approaches, handling of instruments for tumor dissection, as well as application of frequently used technical aids (e.g. surgical microscope, neuro-navigation, intraoperative ultrasound, intraoperative magnetic resonance imaging (MRI), electrophysiological monitoring, and intraoperative fluorescence-guidance). For these reasons, such procedures are typically performed towards the end of the residency and, if at all, with relatively low caseload, as shown recently by a European survey among 532 neurosurgical trainees^9,10^.

A thorough literature search yielded no prior work focusing on the question whether microsurgical intracranial tumor resections, in which a supervised resident is the primary surgeon, achieve comparable complication rates and outcomes to procedures performed by board-certified faculty neurosurgeons (BCFN). We here set out to provide data on this particular research question.

## Results

### Study population and patient demographics

From a total of 1,679 identified cases, 233 were excluded from analysis, as they were exclusively performed by BCFN: 188 transsphenoidal tumor resections (of any entity); 2 chordomas, 30 craniopharyngiomas and 13 pituitary adenomas (resected via craniotomy).

**Figure 1:**
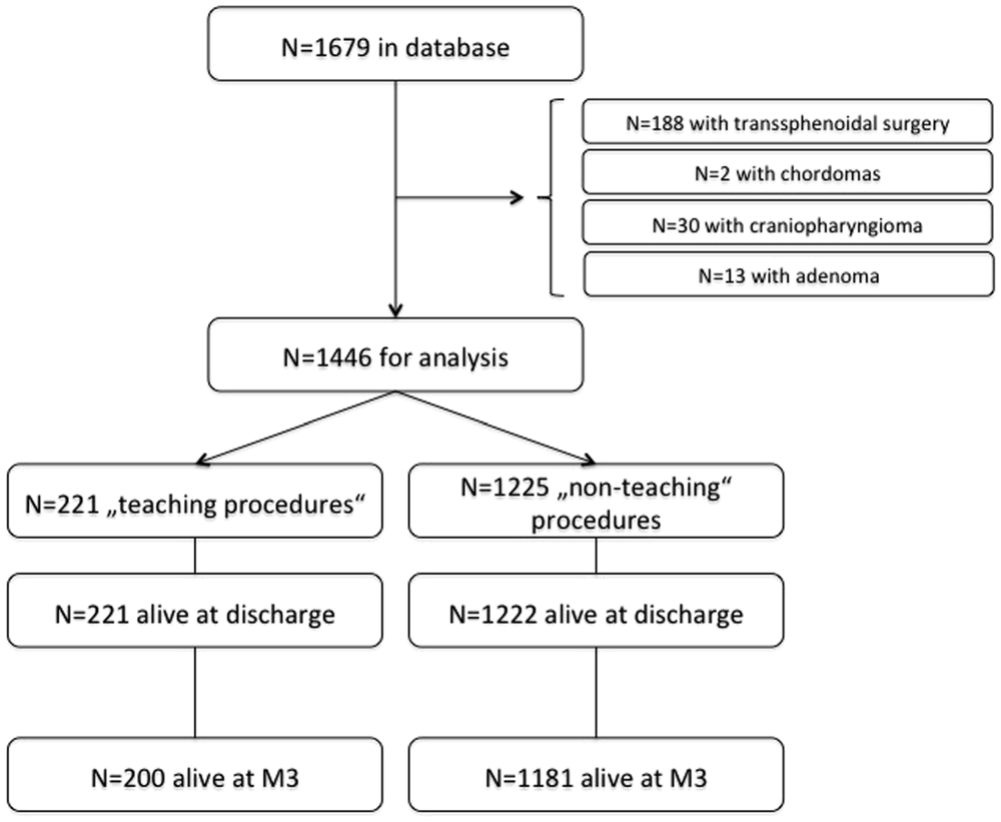
Study algorithm, demonstrating how the final study cohort was arrived at and followed until the 3-month appointment (M3).

Therefore, a total of 1,446 patients (737 female, 51.0%) with a mean age of 54.3 ± 17.3 years (SD) built the final dataset (Fig. 1). 221 (15.3%) procedures were defined as “teaching procedure” in the sense of this research. This group consisted of procedures performed by 27 residents in postgraduate year (PGY) 4 (12.2%), 93 in PGY 5 (42.1%) and 101 in PGY 6 (45.7%). Baseline patient characteristics of both groups are summarized in Table 1. It becomes evident that patients in the teaching group were about 6 years older (p < 0.001), had more comorbidities (American Society of Anesthesiology grading scale; ASA; p = 0.002) and were more often operated on the first time (p = 0.013). The most frequent histopathological diagnoses in the teaching group were metastases (33.0%), followed by glioblastomas (29.9%) and meningiomas (19.0%), whereas in the non-teaching group meningiomas were most frequent (28.1%), followed by glioblastomas (20.5%) and metastases (17.9%; p < 0.001). Non-teaching procedures were more often moderately or highly complex (p < 0.001).

**Table 1:** Baseline patient demographics.

	Teaching procedure	Non-teaching procedure	p-value
Age (in years)	59.3 (14.8)	53.4 (17.6)	<0.001
Sex			
Male	111 (50.2%)	598 (48.8%)	0.700
Female	110 (49.8%)	627 (51.2%)	
ASA class			
1	12 (5.4%)	131 (10.7%)	0.002
2	110 (49.8%)	684 (55.8%)	
3	94 (42.5%)	376 (30.7%)	
4	5 (2.3%)	34 (2.8%)	
Smoking status			
Nonsmoker	128 (57.9%)	735 (60.0%)	0.801
Active smoker	53 (24.0%)	288 (23.5%)	
Former smoker	40 (18.1%)	202 (16.5%)	
Weight (in kg)*	75.7 (17.2)	74.3 (19.3)	0.559
Previous surgery			
Yes	33 (14.9%)	274 (22.4%)	0.013
No	188 (85.1%)	951 (77.6%)	
Type of tumor			
Intraaxial	143 (64.7%)	799 (65.2%)	0.550
Extraaxial	73 (33.0%)	410 (33.5%)	
Unspecified	5 (2.3%)	16 (1.3%)	
Histopathology			
(Epi-)dermoid	2 (0.9%)	16 (1.3%)	<0.001
Anapl. Astrocytoma	5 (2.2%)	89 (7.3%)	
Glioblastoma	66 (29.9%)	251 (20.5%)	
Low grade glioma	11 (5.0%)	144 (11.7%)	
Meningioma	42 (19.0%)	344 (28.1%)	
Metastasis	73 (33.0%)	219 (17.9%)	
Schwannoma	3 (1.4%)	73 (5.9%)	
Other	19 (8.6%)	89 (7.3%)	
Admission KPS			
Good (80–100)	159 (72.0%)	929 (75.8%)	0.386
Moderate (50–70)	53 (24.0%)	261 (21.3%)	
Poor (40–0)	9 (4.0%)	35 (2.9%)	
Admission mRS			
Good (0–1)	113 (51.1%)	694 (56.7%)	0.314
Moderate (2–3)	96 (43.5%)	471 (38.5%)	
Poor (4–5)	12 (5.4%)	60 (4.9%)	
Admission NIHSS			
0–1	148 (67.0%)	844 (68.9%)	0.663
2–5	64 (29.0%)	322 (26.3%)	
≥6	9 (4.0%)	59 (4.8%)	
Tumor size (cm)	3.80 (1.81)	3.72 (1.86)	0.607
Milan Complexity Score			
Low complexity (0–2)	126 (57.0%)	473 (38.6%)	<0.001
Moderate complexity (3–5)	90 (40.7%)	545 (44.5%)	
High complexity (6–8)	5 (2.3%)	207 (16.9%)	
	n = 221 (100%)	n = 1,225 (100%)	

Data is presented in mean (standard deviation) or count (percent). *Indicates an incomplete dataset.

### Analysis of the primary endpoint: morbidity at discharge

Worsening by ≥ 10 points on the Karnofsky Performance Scale (KPS) occurred in 313 patients (21.7%) in total at discharge; 43 (19.5%) and 270 (22.0%) in the teaching and non-teaching group, respectively. In univariate analysis, patients in the teaching group were as likely as patients in the non-teaching group to experience discharge morbidity (OR 0.85, 95% CI 0.60–1.22, p = 0.391). In multivariate analysis, adjusted for baseline differences in age, ASA grade, case complexity (Milan Complexity Scale; MCS)^11^, primary surgery and histopathological tumor type, there was no statistically significant difference in discharge morbidity between the two groups (aOR 1.08, 95% CI 0.74–1.58, p = 0.680; Table 2). Sensitivity analyses, including additional analyses with a 20-point cut-off on the KPS, indicated robustness of the model.

**Table 2:** Relationship between teaching procedure and discharge morbidity.

Discharge morbidity	Univariate analysis			Multivariate analysis		
	OR	95% CI	p-value	OR	95% CI	p-value
Teaching procedure	0.85	0.60–1.22	0.391	1.08	0.74–1.58	0.680
Age ≥ 56 years	1.01	0.79–1.30	0.933	1.28	0.96–1.70	0.098
ASA grade (per 1-step increase)	0.89	0.74–1.08	0.230	0.89	0.72–1.09	0.259
Primary surgery	0.92	0.68–1.24	0.580	0.90	0.66–1.23	0.510
MCS grade (per increase in category)	1.80	1.50–2.15	<0.001*	1.80	1.49–2.17	<0.001*
Metastasis	0.60	0.43–0.85	0.004	0.60	0.39–0.92	0.020
Glioblastoma	1.24	0.93–1.66	0.148	1.00	0.69–1.45	0.997
Meningioma	0.74	0.55–1.00	0.051	0.62	0.44–0.88	0.007

Uni- and multivariate logistic regression analysis estimating the relationship between teaching procedure and worsening on the Karnofsky Performance Scale (KPS) by ≥10 points at time of discharge. The multivariate analysis is adjusted for baseline differences in age (stratified by the median), American Society of Anesthesiology (ASA) grading scale, primary surgery, procedure complexity (Milan Complexity Score; MCS) and the most common histopathological subtypes. *Significant after Bonferroni correction for multiple testing.

### Analysis of the secondary endpoints: morbidity and mortality until M3

Worsening by ≥ 10 points on the KPS occurred in 333 patients (23.0%) in total at M3; 56 (25.3%) and 277 (22.6%) in the teaching and non-teaching group, respectively. In uni- (OR 1.16, 95% CI 0.83–1.62, p = 0.376) and multivariate analysis (aOR 1.03, 95% CI 0.72–1.47, p = 0.887), patients in the teaching group were as likely as patients in the non-teaching group to experience 3-month morbidity (Table 3). Again, results were consistent when a 20-point cut-off for the KPS was chosen in sensitivity analyses.

**Table 3:** Relationship between teaching procedure and 3-month morbidity.

3-month morbidity	Univariate analysis			Multivariate analysis		
	OR	95% CI	p-value	OR	95% CI	p-value
Teaching procedure	1.16	0.83–1.62	0.376	1.03	0.72–1.47	0.887
Age ≥ 56 years	1.59	1.24–2.04	<0.001*	1.31	0.98–1.75	0.063
ASA grade (per 1-step increase)	1.47	1.22–1.77	<0.001*	1.15	0.94–1.40	0.188
Primary surgery	0.78	0.58–1.04	0.085	0.69	0.51–0.94	0.020
MCS grade (per increase in category)	1.35	1.13–1.60	0.001*	1.68	1.38–2.04	<0.001*
Metastasis	1.71	1.28–2.27	<0.001*	2.19	1.48–3.26	<0.001*
Glioblastoma	2.43	1.85–3.18	<0.001*	2.65	1.83–3.82	<0.001*
Meningioma	0.33	0.24–0.47	<0.001*	0.56	0.37–0.85	0.006

Uni- and multivariate logistic regression analysis estimating the relationship between teaching procedure and worsening on the Karnofsky Performance Scale (KPS) by ≥10 points at time of 3-month follow-up. The multivariate analysis is adjusted for baseline differences in age (stratified by the median), American Society of Anesthesiology (ASA) grading scale, primary surgery, procedure complexity (Milan Complexity Score; MCS) and the most common histopathological subtypes. *Significant after Bonferroni correction for multiple testing.

In-hospital mortality was n = 3 patients in the series (0.21%) and all three deaths occurred in the non-teaching group (p = 0.461). The calculation of in-hospital mortality risk estimates was impossible with no deaths occurring in the teaching group.

Mortality at M3 was n = 65 (4.5%) in total; 44 (3.6%) in the non-teaching and 21 (9.5%) in the teaching group, respectively (p < 0.001). In univariate analysis, patients in the teaching group were more likely to experience 3-month mortality as patients in the non-teaching group (OR 2.82, 95% CI 1.64–4.84, p < 0.001). The relationship lost statistical significance in multivariate analysis and after Bonferroni correction for multiple testing (aOR 1.82, 95% CI 1.01–3.26, p = 0.044; Table 4). The causes of death of the 21 patients experiencing 3-month mortality in the teaching group are summarized in Supplementary Table 4.

**Table 4:** Relationship between teaching procedure and 3-month mortality.

3-month mortality	Univariate analysis			Multivariate analysis		
	OR	95% CI	p-value	OR	95% CI	p-value
Teaching procedure	2.82	1.64–4.84	<0.001*	1.82	1.01–3.26	0.044
Age ≥ 56 years	3.24	1.80–5.83	<0.001*	1.54	0.81–2.93	0.185
ASA grade (per 1-step increase)	4.05	2.74–6.00	<0.001*	2.88	1.86–4.47	<0.001*
Primary surgery	0.89	0.49–1.61	0.710	0.64	0.34–1.20	0.164
MCS grade (per increase in category)	0.69	0.47–1.00	0.056	0.91	0.57–1.45	0.692
Metastasis	7.17	4.27–12.0	<0.001*	6.09	2.36–15.7	<0.001*
Glioblastoma	1.50	0.87–2.60	0.148	2.77	1.04–7.39	0.041
Meningioma^+^	—	—	—	—	—	—

Uni- and multivariate logistic regression analysis estimating the relationship between teaching procedure and 3-month mortality. The multivariate analysis is adjusted for baseline differences in age (stratified by the median), American Society of Anesthesiology (ASA) grading scale, primary surgery, procedure complexity (Milan Complexity Score; MCS) and the most common histopathological subtypes.* Significant after Bonferroni correction for multiple testing. ^+^386 observations with meningioma eliminated from analysis, as no 3-month mortality occured in meningioma patients.

### Analysis of the secondary endpoints: in-hospital complications and hospital discharge

Any complication until discharge occurred in 417 patients (28.8%); 48 (21.7%) and 369 (30.1%) in the teaching and non-teaching group, respectively (p = 0.011). In the univariate model, patients in the teaching group had a tendency for less in-hospital complications (OR 0.64, 95% CI 0.46–0.91, p = 0.012), but after statistical adjustment for potential confounders the effect diminished (aOR 0.88, 95% CI 0.62–1.27 p = 0.499; Supplemental Table 2).

Both therapy-oriented classification of complications (CDG; Table 5 p = 0.373) and etiology (Table 6 p = 0.581) were comparable between the teaching and non-teaching group, respectively. Patients in the teaching group were as likely to experience a major complication (CDG 3b or higher) as patients in the non-teaching group (aOR 1.57, 95% CI 0.60–4.12, p = 0.358). They were also as likely as patients in the non-teaching group to experience a complication that was considered to have resulted from direct surgical trauma (aOR 0.91, 95% CI 0.52–1.60, p = 0.741).

**Table 5:** Rate and classification of in-hospital complications.

	Teaching procedure	Non-teaching procedure	p-value
Any complication			
No	173 (78.3%)	856 (69.9%)	0.011
Yes	48 (21.7%)	369 (30.1%)	
Complication severity (CDG)			
1	19 (39.6%)	187 (50.7%)	0.373
2	19 (39.6%)	131 (35.5%)	
3a	3 (6.2%)	12 (3.3%)	
3b	6 (12.5%)	23 (6.2%)	
4a	1 (2.1%)	13 (3.5%)	
4b	−(0%)	−(0%)	
5	−(0%)	3 (0.8%)	
	n = 221 (100%)	n = 1225 (100%)	

Therapy-oriented severity classification of complications according to the Clavien-Dindo grading scale (CDG).

**Table 6:** Rate and etiology of in-hospital complications.

	Teaching procedure	Non-teaching procedure	p-value
CSF-related	2 (4.2%)	15 (4.1%)	0.581
Epilepsy	6 (12.5%)	27 (7.3%)	
General medicine	15 (31.3%)	85 (23.0%)	
Hemorrhagic	2 (4.2%)	26 (7.1%)	
Ischemic	4 (8.3%)	38 (10.3%)	
Septic	−(0.0%)	6 (1.6%)	
Traumatic	16 (33.3%)	158 (42.8%)	
Other	3 (6.2%)	14 (3.8%)	
	n = 48 (100%)	n = 369 (100%)	

Etiology of in-hospital complications classified according to Ferroli *et al*.^11^.

### Extent of resection

A complete resection was achieved in 37.6% of teaching and 34.8% of non-teaching procedures. The resection was incomplete in 28.5% and 35.5% of cases, respectively. In further 344 cases, EOR could not reliably be classified (imaging performed >72h after the operation) and in 95 cases no immediate postoperative imaging was available. Overall, the EOR result did not differ between both groups (p = 0.158). Table 7 summarizes the achieved EOR.

**Table 7:** Extent of resection.

EOR	Teaching procedure	Non-teaching procedure	p-value
Gross total resection	83 (37.5%)	426 (34.8%)	0.158
Subtotal resection	63 (28.5%)	434 (35.5%)	
Unclear	62 (28.1%)	282 (23.0%)	
No imaging available	13 (5.9%)	82 (6.7%)	
	n = 221 (100%)	n = 1224 (100%)	

Extent of resection (EOR), as judged by postoperative magnetic resonance imaging.

### Length of surgery, length of hospitalization and discharge location

Mean length of surgery was 232.0 min (SD 109.8) in the teaching and 268.9 min (SD 133.8) in the non-teaching group (student’s t-test: p < 0.001). Once adjusted for baseline group indifferences by MANOVA, there was no significant difference in the length of surgery (p = 0.337).

Patients in the teaching and non-teaching group were discharged after a mean of 7.6 ± 4.2 and 8.3 ± 5.4 days, respectively (student’s t-test: p = 0.067; MANOVA: p = 0.270). A similar proportion of patients were discharged home (71.6% of total cases), to a nursing home (0.8%), to in-patient rehabilitation (26.3%) or to another location (1.2%; p = 0.694; Supplementary Table 3).

### Subgroup analysis: gliomas, metastases and meningiomas

We performed subgroup analyses in 566 patients with high- and low-grade gliomas, of which 82 (14.5%) were operated by supervised trainees. In multivariate analysis, patients with gliomas in the teaching group were as likely as patients in the non-teaching group to experience morbidity at discharge (aOR 1.17, 95% CI 0.68–2.03, p = 0.568) and at M3 (aOR 0.74, 95% CI 0.42–1.28, p = 0.279). They were also as likely to experience 3-month mortality (aOR 2.33, 95% CI 0.81–6.76, p = 0.117). There was no difference in the EOR (p = 0.713).

Of 292 patients with cerebral metastases, 73 (25.0%) were operated by supervised trainees. In multivariate analysis, patients with cerebral metastases in the teaching group were as likely as patients in the non-teaching group to experience morbidity at discharge (aOR 1.49, 95% CI 0.74–3.04, p = 0.265) and at M3 (aOR 1.28, 95% CI 0.72–2.27, p = 0.398). They were also as likely to experience 3-month mortality (aOR 1.78, 95% CI 0.85–3.73, p = 0.125). There was no difference in the EOR (p = 0.831).

Of n = 386 patients with intracranial meningiomas, 42 (10.9%) were operated by supervised trainees. In multivariate analysis, patients with intracranial meningiomas in the teaching group were as likely as patients in the non-teaching group to experience morbidity at discharge (aOR 0.83, 95% CI 0.30–2.28, p = 0.722) and at M3 (aOR 2.31, 95% CI 0.85–6.27, p = 0.099). There was no 3-month mortality in the group of patients with intracranial meningiomas. There was no significant difference in the EOR (p = 0.156).

### Analysis per PGY

There was no significant difference in morbidity at discharge, morbidity and mortality until M3, as well as complications until discharge for patients in the teaching group, taken care of by a supervised resident in PGY 4, 5 or 6 (Supplementary Table 5).

## Discussion

The objective of this study was to investigate whether microsurgical maximum safe resection of intracranial tumors results in similar complication rates and outcomes when performed by supervised neurosurgical residents, as compared to BCFN. The ultimate question behind the research was – albeit necessary – whether patient safety was negatively affected by the surgical training process, which would then force us to reconsider our training program. The issue is increasingly important nowadays, as operating room (OR) exposure of trainees has decreased together with government-enforced working time restrictions^9,10,12^. Resident training requires new strategies, including simulator training^13–15^, but possibly also earlier resident involvement in the OR environment in order to obtain sufficient skills at time of board-certification. Despite growing interest and relevance, exemplified by a number of previous studies on this subject^1–8^, the relationship between surgical training and both complication rates and clinical outcome had so far not been reported for this particular type of surgical procedure.

The most striking findings were that patients in the teaching group were as likely as patients in the non-teaching group to experience functional worsening at time of discharge and until M3. Furthermore, there were no significant differences in mortality until M3, when a conservative statistical approach with Bonferroni correction for multiple testing was applied. Complication rates, types and severity were comparable between the teaching and the non-teaching group. Further indirect surrogate measures of patient care quality such as EOR, length of hospitalization and discharge location were also similar.

The study cohort resembled the typical population of neuro-oncological patients treated at a tertiary referral center, covering a wide range of pathologies and patients in clinical conditions ranging from very good to very poor (Table 1). A dedicated analysis focusing on patients admitted in a functional dependent condition was published beforehand^16^. Study groups were built by dichotomizing all procedures by the experience level of the surgeon who did most and the key parts of the procedure, similar as in prior reports on this subject^1–8^.

By doing so, a number of baseline variables such as sex, smoking status, tumor type and size, and – importantly – clinical admission status (KPS, modified Rankin scale (mRS) and National Institute of Health stroke scale (NIHSS)) were evenly distributed and balanced across the study groups. However, the study groups were subject to a strong selection by age, overall health status (ASA class), previous surgery, and – most distinctly – tumor type and case complexity (Table 1). Those observed differences in relative frequency of operated tumor entities between the two groups is likely due to intrinsic differences between entities with regard to complexity, including factors such as anatomical distribution and growth pattern. A metastasis is usually easier to identify and to distinguish from the surrounding parenchyma, whereas a low-grade glioma typically shows infiltrative, diffuse growth along the white matter tracts, without clear margins. The selection process of adequate “training cases” is necessary and part of our departmental philosophy. It ensures that the neurosurgical trainee, who takes responsibility for an individual patient, has the required skills level to handle the case. Not doing so could potentially lead to patient harm and would be ethically inacceptable. The fact that this selection process can be appreciated in the dataset underlines its validity, but – unfortunately – complicates the interpretation of the results, as we will discuss in more detail below.

We chose a decrease of ≥10 points on the KPS at hospital discharge as the primary endpoint for a number of reasons. First, it is a broadly accepted grading scale for neuro-oncological patients^17^, and it immediately and reliably reflects outcome after this particular type of surgical treatment with a considerable rate and variety of complications^18,19^. Deliberately choosing time points of outcome assessment relatively soon before and after the surgery allowed us to estimate the direct effect of the studied intervention (operation by supervised trainee) on the outcome, without the natural disease course interfering. Second, it allowed us to apply multivariate logistic regression analysis and calculate robust estimates of the relationship between surgical training and the endpoint, attempting to minimize the effect of the mentioned selection bias by statistical adjustment. Third, prospectively collected information on the KPS was available in all patients without missing data.

In the unadjusted model, the odds for discharge morbidity in patients from the teaching group were lower than 1.0 (OR 0.85, 95% CI 0.60–1.22, p = 0.391), most likely an effect of the lower average case-complexity. This risk estimate changed substantially once adjusted for baseline group differences (aOR 1.08, 95% CI 0.74–1.58, p = 0.680). Since the point estimate is close to 1.0, the 95% CIs are narrow and both subgroup and sensitivity analyses indicated robustness of the model, we can confidently exclude a harmful effect of surgical training on this outcome metric. Of note, the only variable that turned out to be an independent predictor of discharge morbidity was increase in the MCS category; a finding that indicates external validity of this recently proposed grading scale^11^.

In order to rule out any hazardous effect of surgical training on the longer-term outcome, we analyzed 3-month morbidity, for which data was available in all patients. In the unadjusted model, the odds for M3 morbidity in patients from the teaching group were higher than 1.0 (OR 1.16, 95% CI 0.83–1.62, p = 0.376), likely an effect resulting from the differences in histopathological tumor types. The teaching group included twice as many patients with metastases and 10% more patients with glioblastomas, whereas patients harboring benign lesions were more common in the non-teaching group (Table 1). The risk estimate was thus corrected downwards, once adjusted for baseline group differences (aOR 1.03, 95% CI 0.72–1.47, p = 0.887). Again, with a point estimate close to 1.0, relatively tight CIs, and concordant subgroup analyses we can exclude a harmful effect of surgical training on this outcome metric. Besides MCS category, the histopathological tumor types were strong and independent predictors of M3 morbidity, with metastases and glioblastomas significantly increasing and meningiomas decreasing the odds (Table 3).

The perioperative in-hospital mortality rate in this series was 0.2%, and there was no in-hospital mortality in patients from the teaching group (p = 0.461). Our in-hospital mortality rate is slightly below other published reports with perioperative mortality rates ranging from 0.9–2.9% after the resection of intracranial tumors^20–23^. However, it is important to note that a number of limitations exist when comparing mortality rates across different institutions, such as differences in the study population, definition of the perioperative time period and the limited sample size of many previous studies^24,25^.

At the M3 follow-up, mortality in the series had increased to 4.5%. In the unadjusted model, patients from the teaching group had a significantly higher risk of M3 mortality than patients from the non-teaching group (OR 2.82, 95% CI 1.64–4.84, p < 0.001). This effect, however, derives from the distinct differences in histopathological tumor types. It can be appreciated from Table 4 that patients with metastases displayed an impressive excess-mortality, with a six fold increase in M3 mortality risk compared to patients with other tumor types (aOR 6.09, 95% CI 2.36–15.7, p < 0.001). Metastases were the most frequently treated histopathological tumor type of the teaching group, and previous literature reports a median survival ranging from 5–14 months (depending on the primary tumor), and a perioperative mortality of up to 9.6% after cerebral metastasis resection^26,27^. In contrast, there was no M3 mortality in our meningioma patients – the most frequently treated histopathological tumor type of the non-teaching group (Table 1) – with studies portraying a much less aggressive natural course with a 10-year survival rate of 57.4%^28^. Furthermore, as mentioned above, Glioblastoma, again a tumor with a particularly dismal prognosis, was significantly more common in the teaching group^28^.

Accordingly, the effect size of the relationship between surgical training and M3 mortality was noticeably corrected downward, and the finding lost statistical significance with Bonferroni correction for multiple testing (aOR 1.82, 95% CI 1.01–3.26, p = 0.044). As effect of lesser events and the statistical adjustment, the 95% CIs are not as narrow as before, indicating that the point estimate for this relationship may also be less accurate. We therefore reviewed all files of patients from the teaching group with M3 mortality, in order to investigate whether any of the deaths were attributable to the operation/adverse events throughout resident patient care. The cause of death was natural course with tumor progression in 19 (90.4%), pulmonary embolism and unknown in each one (4.7%) patient of the training cohort (Supplementary Table 4). No teaching case was identified where the death was a direct consequence of the supervised trainee operation.

Conducting separate analyses in the subgroups of patients with the two most frequent histopathological tumor types and M3 mortality (gliomas & metastases) allowed us to exclude any selection bias from the tumor type variable *a priori*. These subgroup analyses revealed similar odds for M3 mortality in teaching and non-teaching cases, however with the expected wider 95% CIs and reduced statistical power, compared to the main model. Taking into consideration all these aspects, we can exclude a harmful effect of surgical training on mortality up to M3.

Any deviation from a normal postoperative course was prospectively and systematically recorded in our patient registry^29^. This is important, as the retrospective assessment of complications must rely on accuracy and thoroughness of charting. It has thus been found erroneous and underestimating the true complication incidence^30,31^.

In this cohort, patients in the non-teaching group showed a higher raw complication rate compared to patients in the teaching group (Table 5). Accordingly, in an unadjusted model, teaching procedures tended to have lower odds for complications (OR 0.64, 95% CI 0.46–0.91, p = 0.012). Again, this effect is likely to result from selection bias from case complexity and histopathological tumor type. Hence, in the adjusted model the effect size was corrected upward (aOR 0.88, 95% CI 0.62–1.27, p = 0.499). With a point estimate close to 1.0 and narrow 95% CI, it can be stated that the likelihood to experience an in-hospital complication is similar for teaching and non-teaching procedures.

The severity of complications was analyzed by the CDG, indicating the type of treatment and resource required to deal with a complication (Supplemental Table 1). As can be appreciated from Table 5, there was no difference in the CDG type of complication (p = 0.373). Over 80% of complications in both groups were minor (CDG 1, 2 & 3a), thus not requiring any type of invasive treatment under general anesthesia. As reported in the results section, the adjusted likelihood for complications requiring revision surgery under general anesthesia (CDG 3b), intensive or intermediate care unit management (CDG 4) or resulting in death (CDG 5) was similar for teaching and nonteaching procedures.

The leading etiology of complications in both groups was traumatic (presumably resulting as direct consequence from surgical manipulation), followed by general medicine-related, ischemic, epileptic and hemorrhagic. The noticeably higher incidence of “traumatic” complications in the non-teaching group was most likely a result of the higher case-complexity. After statistical adjustment, the likelihood for traumatic complications was similar for teaching and nonteaching procedures (aOR 0.91, 95% CI 0.52–1.60, p = 0.741).

Extent of resection (EOR) is of critical importance in surgical neuro-oncology, as higher EOR has been linked to better long-term outcomes for a variety of histopathological tumor types^32–37^. Here, one of the main challenges is finding the optimal balance between EOR on the one hand and the functional patient outcome on the other hand. Especially for patients with eloquent tumors or lesions in the vicinity of or surrounding delicate structures (e.g. cranial nerves, major blood vessels), a surgeon may opt for subtotal resection in order to not jeopardize neurologic outcome. In theory, with improving technical skills of a surgeon over time, a greater EOR can be achieved while preserving function.

We collected EOR data for this project in order to rule out that a similar functional outcome had been achieved in the teaching group at the cost of higher rate of residual tumor. Gross total resection was achieved in approximately 37% and 35% of patients and subtotal resection in about 29% of teaching and 35% of non-teaching procedures (p = 0.158). In about a third of procedures in both groups, the EOR was either unclear or impossible to determine. In order to account for the selection bias, we performed subgroup analyses for the three main histopathological tumor types (gliomas, metastases and meningiomas), showing similar EOR in each subgroup.

Prior works of our group and others have found length of surgery to be longer in teaching procedures^1–4,8,38–40^, whereas this finding could not be reproduced in the current series. In the unadjusted analysis, non-teaching procedures had even longer operation times, but these results must again be interpreted with caution. Many factors that were not included in the present analysis may influence length of surgery, e.g. use of intraoperative MRI which was twice as frequently used in the non-teaching group (13.5% vs. 6.2%, p = 0.002). From clinical experience, there is little doubt that neurosurgical trainees are less efficient and – often – even more careful during tumor dissection, which inevitably translates into increased length of surgery. In order to avoid high blood loss and associated complications we therefore often decide for “BCFN backup” in teaching procedures of tumor resections for lesions that are particularly large and/or heavily perfused. The present data do not allow us to underpin our observation of increased length of surgery in teaching procedures with actual numbers. Taking into consideration the previously demonstrated similarity in functional outcome and in-hospital complications, it is not surprising that length of hospitalization was similar. This, but also the similarity in discharge location (e.g., need for inpatient rehabilitation) indicate analogy in quality of patient care between both study groups.

This analysis grounds on the prospective collection of high-quality standardized data from a large cohort of consecutively treated patients that allowed for calculating robust estimates with sufficient power. The inclusion of multiple variables such as clinical outcomes, complications and others, which serve as direct or indirect measures for safety and quality of care, constitute a further strength, as they consistently indicate the same result. Despite its retrospective design, the statistical analyses were predefined, adhering to previously conducted similar studies^1–8^.

There are a number of potential drawbacks of this study, and the most important one is the strong selection bias with implications on the results, as discussed above. This selection required us to use methods for statistical adjustment, which can partially but never completely account for an uneven distribution between study groups. From a scientific point of view, a trial prospectively randomizing “non-complex” patients into either a teaching- or non-teaching group would be best to investigate whether surgical training is safe. However, unless retrospective cohort studies provide some data on this research question first, such a trial may be considered unethical and would likely fail to pass the evaluation of an institutional review board. Taking the results of this study into consideration (difference of 4% in the primary endpoint), a randomized trial would require enrolling 1497 patients per group in order to demonstrate a significant difference between groups. While it is unlikely that such a trial is going to be conducted, we encourage further single- or multicenter reports to provide additional data and challenge our results.

From a methodological point of view, the inclusion of multiple outcomes can be regarded both as strength and weakness. Other than in studies where one single “gold standard” outcome measure exists, it was necessary to investigate the relationship between surgical training and patient outcome from a number of different angles, analyzing multiple relevant variables. For this reason, a considerable number of statistical tests were run, necessitating the use of Bonferroni correction in order to rule out type-I errors.

While there is evidence suggesting that trainee participation in some types of emergency general surgery can be detrimental to patient outcome^41^, our study shows that microsurgical brain tumor resection can be safely performed by supervised neurosurgical residents that are judged capable for the individual case by the team of faculty neurosurgeons.

The strong correlation of case complexity with clinical outcome, as measured by a number of parameters such as morbidity at discharge or complication rate, underlines the need to carefully evaluate and appropriately allocate each case to a neurosurgeon according to his or her skill level. Our results show that it is possible to successfully strike this balance, allowing residents to be in charge of selected cases without putting the patient at additional risks.

While the complexity of intracranial tumor resection can be quantified to some degree by scales such as the MCS, doing the same with regard to a resident’s skill level is much more difficult, making the adequate allocation of cases a subjective process that requires intuition. It is conceivable that in the near future performance models using virtual reality may help with the objective assessment of trainee surgical performance^42^, providing guidance to senior surgeons for the case allocation process and allowing for focused and intensified supervision whenever necessary. More importantly, incorporating innovative teaching techniques such as virtual reality training into structured residency programs may alleviate the challenging task of providing adequate neurosurgical training and exposure to cases in a setting of working hour limitations^42,43^.

## Conclusions

Supervised residents in 4^th^–6^th^ year of training can safely perform craniotomies and microsurgical tumor resections in selected cases, without increasing the risks for mortality, morbidity or complications, when compared to board-certified faculty neurosurgeons. Appropriate allocation of operations according to case complexity and the resident’s experience level, however, appears essential.

## Methods

### Study design and patient identification

The present work is a retrospective, observational cohort study. The prospective database of our department^29^ was searched for consecutive patients who underwent maximum safe microsurgical resection of intracranial tumors between 01/2013 and 12/2017. Patients undergoing diagnostic biopsies were excluded.

### Surgical technique and patient management

Patients are thoroughly examined at admission, and preoperative MRI studies are always requested to plan the surgical strategy; computed tomography (CT) studies only in case of contraindications for MRI or emergencies. For the operation, the surgical microscope, neuro-navigation, electrophysiological neuro-monitoring (motor and sensory evoked potentials, cortical/subcortical stimulation) are frequently applied. We routinely use ultrasonic aspirators for tumor resection and intraoperative ultrasound as well as 3-Tesla intraoperative MRI in select cases to check for resectable tumor remnants^44,45^. Positioning and craniotomies are chosen on a case-by-case basis, depending primarily on the location and extension of the lesion. It is the philosophy of our department that surgery is done without the use of retractors, however, which residents have to apply during teaching procedures, as well.

Postoperatively, patients are usually immediately extubated and kept on the neurosurgical intensive care or intermediate care unit for one night. After MRI studies within the first three postoperative days and transfer to the general ward, the patients are discharged home as soon as they are stable and independent enough. Inpatient rehabilitation is requested for patients with higher-grade motor or otherwise functionally disabling neurological or cognitive deficits.

Outpatient follow-up is routinely performed at three months postoperative (M3) and continued on an individual basis afterwards.

### Study groups and education program

As in previous studies, dichotomization was performed for “teaching procedure” (patient operated on by a supervised neurosurgical resident in PGY 4–6) vs. “non-teaching procedure” (patient operated on by a BCFN)^1–8^. For each “teaching procedure”, one specific BCFN served as trainer, who would discuss the surgical strategy with the resident before the operation. This BCFN would effectively be responsible for the patient from a medico-legal point of view and decide, based on the skill and experience level of the resident, what type of supervision was needed: a) scrubbing in for the operation, b) being present in the OR but not scrubbed-in or c) not being present in the OR, but accessible, if needed. For study purpose, we did not discriminate between the type of supervision, as this information was not available for all cases. In line with previous studies^1–8^, procedures were only classified as “teaching procedures” when most and the major parts of the procedure were independently and completely performed by the resident. If significant parts of the operation were performed by the BCFN, even though the craniotomy or approach might have been performed by the resident, the procedure was labeled a “non-teaching procedure”.

At our department, residents are usually allowed to perform supervised craniotomy and maximum safe resection of brain tumors after having observed a minimum of 50 procedures, from their 4^th^ year of training onwards (PGY ≥ 4), and once considered capable by the team of faculty neurosurgeons. It is an essential part of our training program that relatively straightforward cases are handled by trainees in their “senior resident years” (PGY 5 and 6). The surgeon’s level of training is disclosed to patients and/or their next-of-kin preoperatively during the informed consent. In case a patient does not want to be taken care of by a supervised resident, the patient’s wish is respected (but this is rarely encountered).

For this study, eligible patients were recruited during on-call or at outpatient consultations. Study group assignment was influenced by three factors: 1) according to the hospital’s policy, patients with (semi-)private insurance are always treated by a BCFN; 2) patients that were previously treated by a BCFN are commonly re-operated by the same surgeon; 3) if the case was judged as too difficult for the resident, the procedure was completely or at large parts done by the BCFN (and thus declared a “non-teaching procedure” for the study purpose). While the case allocation process is not standardized, the BCFN responsible for a case decides whether a resident has the necessary competence to be assigned as the primary surgeon based on a number of factors, such as previous experience with similar cases, surgical skills and anatomical knowledge. The department’s senior consultants double-check that case-assignment is adequate on daily radiology reports, where the next days’ surgical cases are presented by the involved resident. Other factors did not influence study group assignment.

### Data collection

All data was withdrawn from the department’s prospective database of complications and outcome^29^. We considered patients’ baseline characteristics including age, sex, American Society of Anesthesiology (ASA) grading scale of perioperative risk, smoking status, weight, previous surgery, functional status (estimated by the KPS, mRS and NIHSS). All of our physicians are instructed in the use of a standardized operating procedure (SOP) for data entry. Furthermore, all are certified for patient evaluation by means of standardized metrics (KPS, mRS, etc.). A study nurse is dedicated to ensuring complete and accurate data collection and certification. Moreover, data quality is reviewed in monthly team meetings.

Disease- and research-specific data that is not part of the registry was added by reviewing electronic patient records. As disease-specific variables, besides the histopathological diagnosis, we recorded maximal diameter (in cm, measured in preoperative imaging) as well as location of the lesion. To estimate the complexity of the case, we computed the Milan Complexity Score (MCS), as previously defined by Ferroli *et al*., stratified into low (MCS 0–2), moderate (3–5) or high complexity (6–8)^11^.

Outcomes were the functional patient condition (KPS) at discharge and M3, mortality at discharge and M3, the rate of in-hospital complications, as well as their therapy-oriented severity (according to the Clavien-Dindo grading scale; CDG; Supplemental Table 1)^46^ and etiology (according to Ferroli *et al*.)^11^.

To determine the extent of resection (EOR), an independent person not involved in the patient care and blinded for the study group allocation reviewed the neuroradiologists written reports of postoperative MRI. EOR was coded as either gross total resection (i.e. no residual tumor), subtotal resection (i.e. residual tumor of any size), unclear (e.g. MRI conducted > 72 h after the operation or neuroradiologist unsure concerning the presence of residual tumor) or no immediate postoperative MR-imaging available.

Length of surgery (in minutes), length of hospitalization (in days) and discharge location (home; nursing home; rehabilitation/other hospital; other) were used as surrogate markers for complications and outcome.

### Statistical considerations

Demographic baseline data were described using frequencies and percentage for categorical variables. Interval-scaled variables were described as means and standard deviations (SD), respectively. Imbalances between the dependent and independent groups were tested using Pearson χ^2^ tests, student’s t-tests and multivariate analysis of variance (MANOVA), as appropriate.

The primary endpoint was morbidity at discharge, defined as postoperative worsening of ≥10 points on the KPS, compared to the preoperative admission grading. We deliberately chose the 10-point difference as cut-off^11^ in order not to overlook subtle differences in the functional status. From here, logistic regression analysis allowed for estimation of the effect size of the relationships between “teaching procedure” and the outcomes of interest. First, univariate models were built to calculate direct relationships. Then, multivariate models were adjusted for all baseline characteristics that were found to differ between the study groups, as determined in Table 1. The (adjusted) odds ratios ((a)OR) and 95% confidence intervals (CI) were analyzed for changes, and both subgroup and sensitivity analyses were made.

Secondary endpoints were morbidity at M3, mortality at discharge and M3, rate, severity and etiology of in-hospital complications, besides EOR, length of hospitalization and discharge location.

As there were important group heterogeneities, we preferably created binary variables to allow for statistical adjustment. For categorical variables, however, group-differences were tested using unadjusted Pearson χ^2^-tests. With 28 statistical tests run for the analysis of the main outcomes and to account for multiple testing by Bonferroni correction, p-values < 0.0018 (=0.05/28) were regarded as statistically significant. All analyses were performed with Stata version 14.2 for Mac (College Station, TX: StataCorp LP).

### Sample size calculation and statistical power

This study was retrospective, and therefore primarily limited by the number of available datasets in the institutional patient registry. However, in order to detect a significant difference of 10% in the primary endpoint, 165 per group would be required with a power of 0.80 and alpha set at 0.05.

### Ethical considerations

The institutional review board “Kantonale Ethikkommission Zürich” approved the workup of registry data (KEK-ZH 2012-0244) and the protocol was registered at http://www.clinicaltrials.gov (Identifier: NCT01628406). Informed consent was obtained from adult participants included in the study and from a parent and/or legal guardian for participants under the age of 18 years. The study follows the STROBE recommendation for observational studies.

## Supplementary information

Supplemental Material
