# Supplementary material for: Safety of resident training in the microsurgical resection of intracranial tumors: Data from a prospective registry of complications and outcome
Source: Sci Rep. 2019 Jan 30;9:954. doi: 10.1038/s41598-018-37533-3 (PMC6353994; doi:10.1038/s41598-018-37533-3)
Supplement: Supplementary file 1 — Supplemental Material [file 41598_2018_37533_MOESM1_ESM.pdf]

Supplemental Material

Associated with the article:

Safety of resident training in the microsurgical resection of intracranial tumors: Data from a prospective registry of complications and outcome

Flavio Vasella[1,2*], MD; Julia Velz[1,2*], MD; Marian C. Neidert[1,2], MD; Stephanie Henzi[1,2], MMed; Johannes Sarnthein[1,2], PhD; Niklaus Krayenbühl[1,2], MD; Oliver Bozinov[1,2], MD; Luca Regli, MD; Martin N. Stienen[1,2], MD, FEBNS

[1]*University Hospital Zurich, Department of Neurosurgery, Zurich, Switzerland*

[2]*Clinical Neuroscience Center, University of Zurich, Zurich, Switzerland*

[*]Both FV and JV have contributed equally as first authors to this work.

Supplemental Table 1. The Clavien-Dindo grading scale (CDG) for the severity of complications.

**CDG**	**Definition**
Grade 1	Any deviation from the normal postoperative course without the need for pharmacological treatment or surgical, endoscopic, and radiological interventions. Acceptable therapeutic regimens are: drugs as antiemetics, antipyretics, analgetics, diuretics and electrolytes and physiotherapy. This grade also includes wound infections opened at the bedside.
Grade 2	Requiring pharmacological treatment with drugs other than such allowed for grade I complications. Blood transfusions and total parenteral nutrition are also included.
Grade 3	Requiring surgical, endoscopic or radiological intervention
Grade 3a	Intervention not under general anesthesia
Grade 3b	Intervention under general anesthesia
Grade 4	Life-threatening complication requiring intensive care/intensive care unit management
Grade 4a	Single organ dysfunction (including dialysis)

Grade 4b	Multi organ dysfunction
Grade 5	Death of a patient

Supplemental Table 2: Uni- and multivariate logistic regression analysis estimating the relationship between teaching procedure and occurrence of any in-hospital complication. The multivariate analysis is adjusted for baseline differences in age (stratified by the median), American Society of Anesthesiology (ASA) grading scale, primary surgery, procedure complexity (Milan Complexity Score; MCS) and the most common histopathological subtypes.

**Complication**	**Univariate analysis**			**Multivariate analysis**		
	**OR**	**95% CI**	**p-value**	**OR**	**95% CI**	**p-value**
Teaching procedure	0.64	0.46 – 0.91	0.012	0.88	0.62 – 1.27	0.499
Age ≥ 56 years				1.09	0.84 – 1.42	0.510
ASA grade (per 1-step increase)				1.13	0.93 – 1.36	0.217
Primary surgery				1.06	0.79 – 1.43	0.677
MCS grade (per increase in category)				1.78	1.50 – 2.12	<0.001*
Metastasis				0.38	0.25 – 0.57	<0.001*
Glioblastoma				0.77	0.54 – 1.08	0.132
Meningioma				0.91	0.67 – 1.24	0.552

* Significant after Bonferroni correction for multiple testing.

Supplementary Table 3: Discharge location in patients operated on by supervised trainees (teaching procedures) and board-certified faculty neurosurgeons (non-teaching procedures).

	**Teaching procedure**	**Nonteaching procedure**	**p-value**
Home	165 (74.6%)	870 (71.0%)	0.694
Nursing home	2 (0.9%)	10 (0.8%)	
Rehabilitation / Clinic	51 (23.1%)	330 (27.0%)	
Other	3 (1.4%)	15 (1.2%)	
	**n = 221 (100%)**	**n = 1225 (100%)**	

Supplementary Table 4: Cause of death in patients operated on by supervised trainees (teaching procedures) with early mortality within 3 months after surgery. ALL = Acute Lymphoblastic Leukemia; F = Female; GBM = Glioblastoma; M = Male; MTX = Metastasis.

Case number	Age in years	Sex	Diagnosis	Cause of death
1	66	M	GBM	GBM progression / Palliative care
2	68	M	GBM	GBM progression / Palliative care
3	72	M	GBM	GBM progression / Palliative care
4	69	F	MTX	Tumor progression / Palliative care
5	69	F	MTX, ALL	ALL progression / Palliative care
6	54	M	MTX	Tumor progression / Palliative care
7	60	F	MTX	Tumor progression / Palliative care
8	60	F	MTX	Tumor progression / Palliative care
9	53	F	MTX	Tumor progression / Palliative care
10	73	F	MTX	Tumor progression / Palliative care
11	79	F	GBM	GBM progression / Palliative care
12	71	F	MTX	Fulminant pulmonary embolism; unsuccessful cardio-pulmonary resuscitation in external hospital
13	41	M	MTX	Tumor progression / Palliative care
14	68	F	MTX	Tumor progression / Palliative care
15	84	M	MTX	Cause of death unclear
16	71	F	GBM	GBM progression / Palliative care
17	74	F	MTX	Tumor progression / Palliative care
18	71	M	MTX	Tumor progression / Palliative care
19	64	F	GBM	GBM progression / Palliative care
20	61	M	MTX	Tumor progression / Palliative care
21	86	F	MTX	Tumor progression / Palliative care

Supplementary Table 5: Cause of death in patients operated on by supervised trainees (teaching procedures) with early mortality within 3 months after surgery. PGY = postgraduate year.

Outcome	Training year			Total	p-value
	PGY 4	PGY 5	PGY 6		
Discharge morbidity	3 (11.1%)	22 (23.7%)	18 (17.8%)	43 (19.5%)	0.298

3-month morbidity	8 (29.6%)	24 (25.8%)	24 (23.8%)	56 (25.3%)	0.816
3-month mortality	4 (14.8%)	10 (10.8%)	7 (6.9%)	21 (9.5%)	0.400
Discharge complication	7 (25.9%)	23 (24.7%)	18 (17.8%)	48 (21.7%)	0.432
	**n=27 (100%)**	**n=93 (100%)**	**n=101 (100%)**	**n=221 (100%)**	
